# Transmission dynamics of pandemic influenza A(H1N1)pdm09 virus in humans and swine in backyard farms in Tumbes, Peru

**DOI:** 10.1111/irv.12329

**Published:** 2015-12-11

**Authors:** Yeny O. Tinoco, Joel M. Montgomery, Mathew R. Kasper, Martha I. Nelson, Hugo Razuri, Maria C. Guezala, Eduardo Azziz‐Baumgartner, Marc‐Alain Widdowson, John Barnes, Robert H. Gilman, Daniel G. Bausch, Armando E. Gonzalez

**Affiliations:** ^1^ U.S. Naval Medical Research Unit No. 6LimaPeru; ^2^ Johns Hopkins School of Public HealthBaltimoreMDUSA; ^3^ U.S. Centers for Disease Control and PreventionDivision of Global Health ProtectionNairobiKenya; ^4^ Fogarty International CenterNational Institutes of HealthBethesdaMDUSA; ^5^ U.S. Centers for Disease Control and PreventionAtlantaGAUSA; ^6^ Tulane School of Public Health and Tropical MedicineNew OrleansLAUSA; ^7^ San Marcos UniversityVeterinary SchoolLimaPeru

**Keywords:** Antibodies, backyard pig farms, human–animal transmission, influenza

## Abstract

**Objectives:**

We aimed to determine the frequency of pH1N1 transmission between humans and swine on backyard farms in Tumbes, Peru.

**Design:**

Two‐year serial cross‐sectional study comprising four sampling periods: March 2009 (pre‐pandemic), October 2009 (peak of the pandemic in Peru), April 2010 (1st post‐pandemic period), and October 2011 (2nd post‐pandemic period).

**Sample:**

Backyard swine serum, tracheal swabs, and lung sample were collected during each sampling period.

**Main outcome measures:**

We assessed current and past pH1N1 infection in swine through serological testing, virus culture, and RT‐PCR and compared the results with human incidence data from a population‐based active surveillance cohort study in Peru.

**Results:**

Among 1303 swine sampled, the antibody prevalence to pH1N1 was 0% pre‐pandemic, 8% at the peak of the human pandemic (October 2009), and 24% in April 2010 and 1% in October 2011 (post‐pandemic sampling periods). Trends in swine seropositivity paralleled those seen in humans in Tumbes. The pH1N1 virus was isolated from three pigs during the peak of the pandemic. Phylogenetic analysis revealed that these viruses likely represent two separate human‐to‐swine transmission events in backyard farm settings.

**Conclusions:**

Our findings suggest that human‐to‐swine pH1N1 transmission occurred during the pandemic among backyard farms in Peru, emphasizing the importance of interspecies transmission in backyard pig populations. Continued surveillance for influenza viruses in backyard farms is warranted.

## Introduction

The 2009 influenza pandemic emphasized the continued threat that zoonotic influenza viruses pose to global health. Avian and swine influenza viruses present a particular threat to humans because of their potential for reassortment and emergence of genetically novel strains.^1^ In particular, influenza viruses of swine origin have demonstrated their pandemic potential.^2^ The influenza A(H1N1)pdm09 virus (pH1N1) was first identified among humans in March 2009 and generated the first pandemic of the 21st century.^3^ Since 2009, pH1N1 has become endemic in human populations globally and there have been numerous reports of human‐to‐swine transmission.^1^, ^4^, ^5^, ^6^, ^7^, ^8^, ^9^, ^10^, ^11^, ^12^, ^13^, ^14^, ^15^, ^16^, ^17^, ^18^, ^19^, ^20^ Most of these reports, however, have focused on larger scale industrial farms,^4^, ^7^, ^11^, ^18^ as opposed to smaller scale backyard farms, despite the fact that large numbers of pigs are raised in backyard settings, particularly in developing countries, providing considerable opportunity for influenza virus transmission between humans and livestock.^21^, ^22^

In Peru, are no data exist regarding influenza viruses among swine populations. Similar to many low‐ and middle‐income countries, the vast majority of swine (3·8 million, 80%) are found on backyard farms.^23^ We conducted a 2‐year serial cross‐sectional study of the prevalence of pH1N1 infection and past exposure among swine from community backyard farms in northern Peru. In addition, we were able to compare the data on swine with incidence data on human influenza from a population‐based active surveillance cohort study conducted at the same time in the region.^24^

## Methods

### Study settings and design

**Figure 1:**
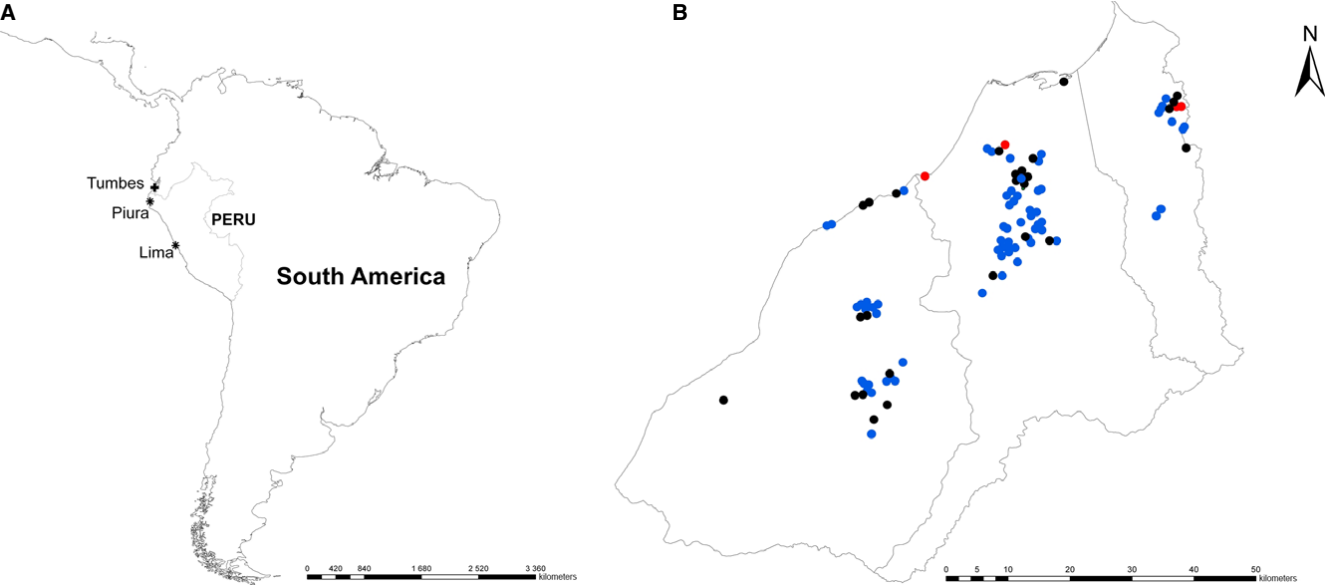
(A) Map of South America showing the study site region (indicated by a cross) and the geographic provenance of the three viruses (indicated by stars) which clustered most closely on phylogenetic analysis with the viruses obtained from swine in Tumbes. (B) Map of the Tumbes region, Peru, showing sites of backyard farms with pigs positive for antibody to influenza A(H1N1)pdm09 virus during the peak pandemic period (black circles), the 1st post‐pandemic period (blue circles), and during the 2nd post‐pandemic pandemic period (red circles).

**Figure 2:**
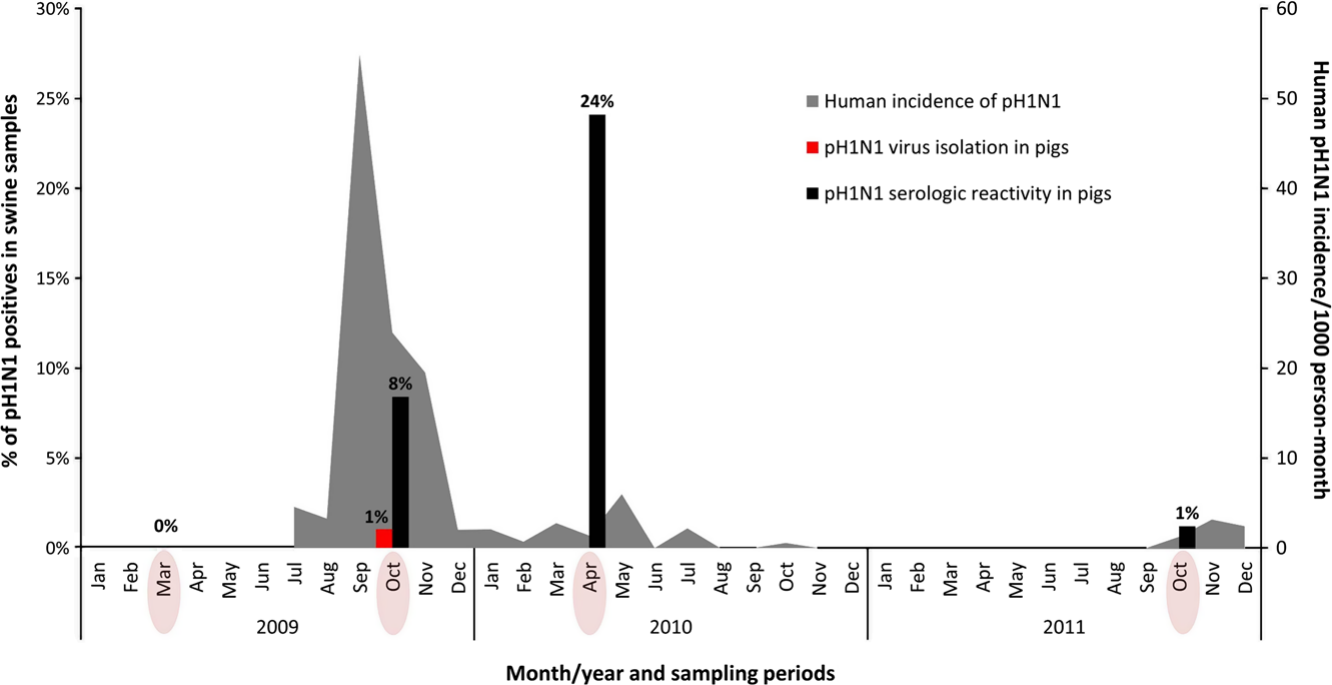
Antibody prevalence and isolation of influenza A(H1N1)pdm09 virus (pH1N1) from sera and tracheal swabs of backyard pigs collected in Tumbes, Peru, 2009–2011. The dates of the four sampling periods are circled on the *x*‐axis. The incidence of pH1N1 influenza in humans in Tumbes is shown in gray, obtained from surveillance through an influenza cohort study conducted by our research team which began on July 27, 2009.^24^ There was no evidence of pH1N1 influenza activity during November 2010 to September 2011. The first case of pH1N1 influenza in humans in Tumbes was reported by the Peruvian Ministry of Health on July 1, 2009.

Tumbes, a region of approximately 210 000 people on the northern coast of Peru, is largely comprised of small semirural communities where pigs and poultry are commonly raised for personal consumption (Figure 1). We conducted a serial cross‐sectional study in Tumbes comprising four sampling periods: March 2009 (pre‐pandemic), October 2009 (peak of the pandemic in Peru), April 2010 (1st post‐pandemic period) and October 2011 (2nd post‐pandemic period) (Figure 2).^25^

### Animals and sample collection

Serum samples for influenza virus testing were collected and made available by the Cysticercosis Elimination Program (CEP) in Peru.^26^ As part of the CEP's yearly surveillance activities in Tumbes, cysticercosis seronegative and seropositive backyard swine were identified by Western blot testing^27^ from sera collected previously and swine were purchased directly from their household owners at each time period as detailed previously.^26^ They were collected from 965 total backyard farms and transferred to the CEP facility where they were kept in pens separated by age and size for 1–5 days before being euthanized and slaughtered. A veterinarian evaluated the animals' general health condition and recorded daily observations. Swine slaughter was conducted under an animal protocol approved for the CEP study.^26^ Sampling and testing for influenza virus were approved by the U.S. Naval Medical Research Unit No. 6 (NAMRU‐6, Lima, Peru) Institutional Animal Care and Use Committee.

At the time of slaughter, in addition to the serum samples obtained, we collected samples of the respiratory tract (trachea and lungs) of pigs. Tracheal samples were obtained by vigorous swabbing of the tracheal wall followed by placement of swabs in viral transport medium.^28^ Lung samples were obtained by biopsying the cranial lobes of each lung, where lesions from pH1N1 infection are most frequent identified,^29^ and placing the tissues in cryovials. Respiratory tract samples were transported at approximately 4°C to the CEP in Tumbes between 30 and 180 minutes following collection where they were stored in liquid nitrogen until sent to the San Marcos University Veterinary School in Lima, Peru, for testing. All samples collected by the CEP were tested for influenza regardless of cysticercosis infection status.

### Human influenza data

Data on incidence of human influenza were from a population‐based active surveillance cohort study begun in 2009 by NAMRU‐6 with support from the Peruvian Ministry of Health, the U.S. Centers for Disease Control and Prevention in Atlanta, GA, and the Armed Forces Health Surveillance Center in Silver Spring, MD.^24^, ^30^ In the study, over 2500 randomly selected households comprising more than 10 000 people in five sites in Peru, including approximately 450 households and 1800 people in Tumbes, were visited up to three times a week to screen household members for influenza‐like illness. Nasopharyngeal swabs were collected from identified cases and tested for influenza viruses by PCR. The study was approved by the NAMRU‐6 Institutional Review Board (NMRCD.2009.0005).

### Laboratory methods

#### Hemagglutination inhibition

Sera were tested for antibodies to pH1N1 by hemagglutination inhibition (HI) assay as previously described^31^ using an influenza virus (A/swine/Peru/2010729235/2009) isolated from a pig in this study via culture in fertilized specific‐pathogen‐free (SPF) chicken eggs. Sera were pre‐treated with receptor‐destroying enzyme (Denka Seiken Co. Ltd., Tokyo, Japan) and heme‐absorbed with turkey erythrocytes. Titer results are reported as the reciprocal of the highest dilution of serum that inhibited virus‐induced hemagglutination of a 0·5% (v/v) solution of erythrocytes. The HI titer was expressed as the highest reciprocal serum dilution that completely inhibited hemagglutination of one hemagglutinin (HA) unit. We chose ≥1:10 as threshold of antibody titer to define seropositivity to pH1N1 infection, based on previous findings^7^ and the finding of absence of HI antibody against pH1N1 in the pre‐pandemic samples.

#### Virus isolation

Virus isolation was conducted by standard methods.^32^ Pools of five individual swabs were made according to date, sample type, and community. Pools were homogenized and filtered before inoculation into the allantoic cavity of five SPF 9‐day‐old embryonated chicken eggs. Eggs were incubated for 6 days with daily survival checks. Allantoic fluid of each egg was tested for hemagglutinating agents by direct HI. Negative pools were passaged a second time to confirm negativity. HI‐positive allantoic fluids were confirmed by antigen presence using the QuickVue Influenza Test (Quidel Corp., San Diego, CA, USA), after which each individual sample was cultured again following similar procedures.

#### Influenza virus PCR, gene sequencing, and analyses

For swine specimens, RNA extracts were prepared from 100 μl of allantoic fluid with the MagNA Pure Compact automated RNA extraction system (Roche Applied Science, Indianapolis, IN, USA). One‐step RT‐PCR (Invitrogen, Carlsbad, CA, USA) was used to amplify HA and neuraminidase (NA) genes with universal HA and NA oligonucleotide primers.^33^ Specimens from humans were tested for influenza virus by RT‐PCR using standard methods.^34^

**Figure 3:**
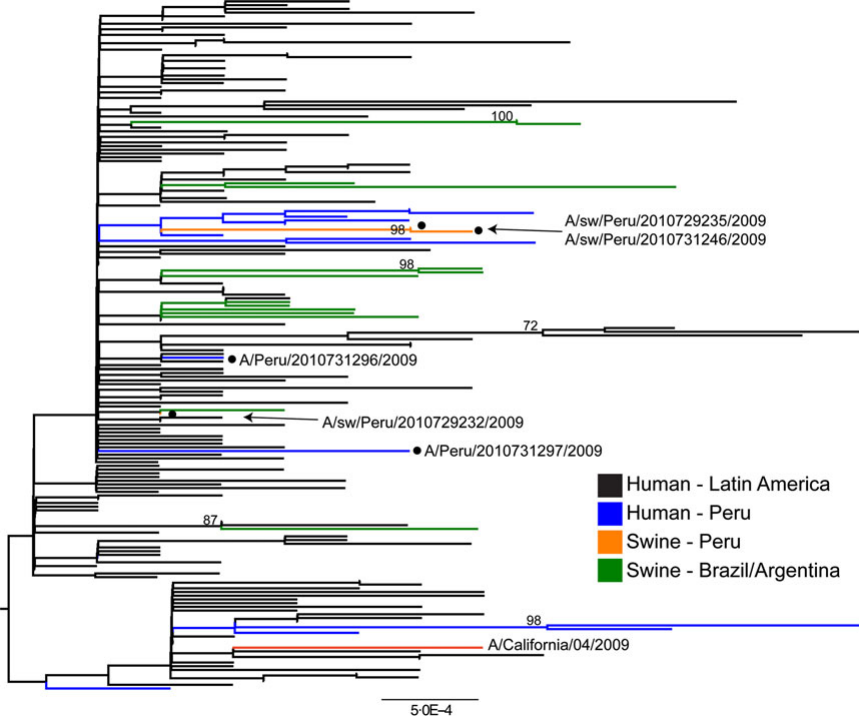
Maximum‐likelihood phylogeny of concatenated hemagglutinin and neuraminidase gene sequences from influenza A(H1N1)pdm09 viruses from Latin America, 2009–2011. Virus sequences shown were collected from three swine (orange with circles) and two humans in Tumbes (blue with circles) (see explanation in text), 13 swine in Argentina and Brazil (green), 14 humans in other sites in Peru (blue), 132 humans in other Latin American countries (black), and the red line: Influenza A/California/07/2009. Bootstrap values >70 are included for key nodes, and tree is midpoint rooted for clarity only. GenBank accession numbers of the sequences used in this analysis are listed in Appendix 2.

Amplicons were purified by agarose gel electrophoresis followed by purification using the MinElute Gel Extraction Kit (QIAGEN, Valencia, CA, USA), and then sequenced on an automated Applied Biosystems 3730 system (Foster City, CA, USA) using cycle sequencing dye terminator chemistry. Ultimately, our study yielded five complete HA sequences (three from pigs in northern Peru, two from humans in northern Peru), five complete NA sequences (three from pigs, two from humans), five complete MP sequences (three from pigs, two from humans), and four complete whole‐genome sequences (two from pigs, two from humans). Whole‐genome sequences from pH1N1 viruses available on GenBank from the Western Hemisphere were included as background. Additional HA and NA sequences from swine in Latin America (in this case, Brazil and Colombia) were added, for which whole‐genome sequences were not available. Separate alignments for each of the eight genome segments were made using ClustalW (v1.83).^35^ As there was no evidence of reassortment between the HA and NA for our data, the HA and NA segments were concatenated^36^ for greater phylogenetic resolution and visual clarity in Figure 3. A maximum‐likelihood (ML) tree was inferred for each segment separately using RAxML v.7.2.6 (GTR+gamma substitution model), with statistical support for individual nodes assessed by bootstrap analysis (500 replicates). GenBank accession numbers of the sequences used in this analysis are listed in Appendix 2.

### Statistical methods

The primary variable and outcome of interest was antibody to pH1N1 (≥1:10) assessed by the HI assay.^7^ We compared antibody prevalence between the 2009 and each other sampling periods using binary logistic regression adjusting for age and gender.

## Results

Over the course of the study, we collected serum samples from 1303 backyard swine (310 pre‐pandemic, 322 peak of the pandemic, 328 1st post‐pandemic period, and 343 2nd post‐pandemic period). Tracheal and lung samples were available for all periods except for the 2nd post‐pandemic period. We collected 923 tracheal swabs and lung samples (310 pre‐pandemic, 288 peak of the pandemic, and 325 1st post‐pandemic period). None of the pigs showed symptoms of respiratory disease. The age of swine ranged from 2 to 60 months, and 75% were aged between 6 and 8 months (Table 1).

**Table 1:** Demographic characteristics of swine sampled in Tumbes, Peru, 2009–2011

Age, months	Sampling period								Total	
	Pre‐pandemic (March 2009)		Peak pandemic (October 2009)		1st Post‐pandemic (April 2010)		2nd Post‐pandemic (October 2011)			
	*n* = 310	(%)	*n* = 322	(%)	*n* = 328	(%)	*n* = 343	(%)	*n* = 1303	(%)
<6	154	(50)	0	(0)	0	(0)	64	(19)	218	(17)
6–8	54	(17)	322	(100)	328	(100)	279	(81)	983	(75)
>8	102	(33)	0	(0)	0	(0)	0	(0)	102	(8)
Sex										
Male	190	(61)	165	(51)	165	(50)	166	(48)	686	(53)

None of the 310 pre‐pandemic serum or respiratory samples was positive for antibodies against pH1N1 or pH1N1 virus (Table 2). The antibody prevalence of swine samples collected during the pandemic was 8% (27/322), 95% CI: 6–12, with pH1N1‐positive virus isolation and RT‐PCR testing from three tracheal swabs [1% (3/288), 95% CI: 0·2–3] and one lung sample [0·3% (1/288), 95% CI: 0–1·9], the latter from one of the animals with a positive tracheal swab (Figure 2). All three virus isolates were from pigs with HI antibody titers <1:10. Additionally, we also evaluated cutoff points of antibody titers <1:40 and observed similar trends, just a change in magnitude of positivity. Twenty‐four percent of samples (79/328, 95% CI: 20–29) were seropositive 6 months following the pandemic peak and 1·2% (4/343), 95% CI: 0·3–3, 2 years following the peak (Figure 2). Even though highest seroprevalence was reported during the 1st post‐pandemic period, antibody titers were highest during the pandemic period (i.e., 48% of positive swine (13/27) had HI titers of 80–160) and decreased progressively during the two post‐pandemic samplings [17% (13/79) and 0% (0/4), respectively] (Table 2).

**Table 2:** Hemagglutination titers against influenza A(H1N1)pdm09 virus in pig sera in Tumbes, Peru, 2009–2011

HI titer^a^	No. of sera positive (%) by period			
	Pre‐pandemic (March 2009)	Peak pandemic (October 2009)	Post‐pandemic 1 (April 2010)	Post‐pandemic 2 (October 2011)
10	0 (0)	3 (11)	16 (20)	1 (25)
20–40	0 (0)	10 (37)	50 (63)	3 (75)
80–160	0 (0)	13 (48)	13 (17)	0 (0)
640	0 (0)	1 (4)	0 (0)	0 (0)
Total	0 (0)	27 (100)	79 (100)	4 (100)

a The limit of detection for the hemagglutination assay was set to ≥1:10.

The HA and NA gene nucleotide sequences from the three pH1N1‐positive animals were ≥99% homologous compared with those of viruses identified during the same time period among humans in Tumbes (Appendix 1). Phylogenetic analysis of the HA and NA segments, however, indicated that the three swine viruses collected in our study and two viruses collected from humans in Tumbes in October 2009 were not monophyletic, indicative of more than one human‐to‐swine introduction (Figure 3). Two of the pH1N1 viruses found in swine (A/swine/Peru/2010729235/2009 and A/swine/Peru/2010731246/2009) clustered together with high bootstrap support on the NA tree (98%), suggesting that they could represent a single human‐to‐swine transmission event with onward transmission in swine (the viruses were collected 2 days apart: October 17, 2009, and October 19, 2009). These viruses were most closely related to human pH1N1 viruses collected from humans from our study in Tumbes.

Despite being closely related phylogenetically, A/swine/Peru/2010729235/2009 and A/swine/Peru/2010731246/2009 were collected on farms in different small communities of Tumbes (approximately 18·5 km apart) (Figure 1). This pair of swine viruses therefore represents one of three scenarios: long‐distance swine–swine transmission of a single viral introduction of human origin, two separate introductions of very similar pH1N1 viruses from humans, or viral transmission within the swine holding facility prior to sampling. The possibility of two separate human‐to‐swine transmissions cannot be ruled out due to the low number of background viruses available from humans in the Tumbes region. The third swine virus (A/swine/Peru/2010729232/2009), although collected at nearly the same time (October 15, 2009), is positioned in a separate part of the tree and likely represents another separate human‐to‐swine introduction within northern Peru (Figure 3).

## Discussion

Our 2‐year cross‐sectional study revealed a high rate of infection of backyard swine in Tumbes, Peru, with pH1N1 viruses of human origin, with a peak of 24% seropositive during April 2010. We found phylogenetic evidence for two likely viral introductions into swine in Tumbes, as well as limited swine‐to‐swine transmission, although it is not clear whether this occurred in the backyard farm setting or subsequently in the swine holding pen. It would be of great interest to determine how extensively pH1N1 spread swine‐to‐swine within these backyard farm settings, or whether the virus mainly was reseeded in pigs via human introductions with little onward transmission in swine. However, such fine‐scale transmission dynamics will require further surveillance and sequencing.

During 2009–2011, pH1N1 infection in pigs on backyard farms closely paralleled pH1N1 activity in humans living in the same communities. The pH1N1 virus was introduced into the swine population within 3 months of the first reported human case in the region by the Peruvian Ministry of Health on July 1, 2009. Similar time lags between human and swine infection have been noted in studies of humans and swine on backyard farms in Vietnam, Sri Lanka, and Cameroon.^5^, ^8^, ^14^ The increase in antibody prevalence (24%) in swine during the first post‐pandemic period, in which most pigs sampled were 6–8 months old, likely represents human‐to‐swine infections that occurred during the peak of the human influenza pandemic and possibly limited onward transmission of pH1N1 in swine. Antibodies against classical H1 swine influenza virus in pigs can last for more than 1 year after primary infection,^37^ and we report the majority of HI titers in pigs were <1:80, 8 months after the peak pandemic period, indicating a decay in antibodies consistent with prior exposure. Additionally, although transfer of maternal antibody typically lasts 1–2 months in pigs, it could also account for some seropositive animals.^38^, ^39^

By the second post‐pandemic period (October 2011), the antibody prevalence in swine had fallen to 1%, indicating that pH1N1 had not become endemic in the swine population in Tumbes. The small number of pigs that remained positive likely represents some latent immunity among a small proportion of swine population or occasional transmission from swine or humans infected with pH1N1 during a period with very low incidence of pH1N1 in humans. Interestingly, the 1% antibody prevalence in swine corresponds to a slight increase in pH1N1 influenza in humans during the second post‐pandemic period at this time (Figure 2).

Our study reports the introduction of human pH1N1 virus into a low‐density backyard swine population in a rural community where influenza viruses have not been previously detected in swine. Our low rate of viral isolation does not allow us to determine whether the majority of swine infections were the result of contact with pH1N1‐infected humans or transmission between swine, although our findings do suggest at least limited transmission in backyard swine. The fact that most of these animals were raised in separate small backyard farms until days before slaughter indicates that influenza viruses can still infect swine in settings with low animal contact rates owing to high contact with humans.

The high incidence of human pH1N1 influenza (up to 55/1000 person‐months) during the peak pandemic period likely afforded ample opportunity for human‐to‐swine transmission. Although experimental infections in the laboratory confirm the potential for pH1N1 virus shedding and contact transmission between swine,^20^, ^40^ most field investigations suggest that sustained transmission among swine is limited in small‐scale farm settings^5^, ^14^ although, as infection is often subclinical or associated with mild disease,^4^, ^9^ it may go undetected.^40^ Consistent with this, none of the swine in our study were noted to be sick prior to slaughter and no abnormalities were noted on gross anatomic examination of the lungs of the three virus‐positive animals. The primary impediment to virus transmission between swine in backyard farms is likely to be more situational than biological, that is, a relatively small number of pigs are raised together, with limited opportunity for contact with animals from other household farms before slaughter. The typically brief period of time that animals are brought together for slaughter is probably inconsequential to transmission between swine, although the potential for swine‐to‐human infection remains.

The fact that the three pH1N1 viruses found in swine in Tumbes were not genetically identical to those found in humans in the region at the same time reflects how rapidly influenza virus disseminates through communities, such that it is difficult to infer the spatial origins of the direct progenitors of the swine viruses detected in our study. Furthermore, the comingling of swine prior to slaughter undermines the ability to assess the geographic origins of all three swine isolates. The most parsimonious explanation is that the swine in our study acquired pH1N1 viruses directly from humans or during close contact with other pigs within the CEP facility pens. However, we cannot rule out the possibility of longer distance transmission. Further isolation and sequencing of viruses from both swine and humans in our study would be required to further delineate routes of transmission.

### Limitations

We used a relatively low HI titer (1:10) to define pH1N1 seropositivity. Nevertheless, as none of the pre‐pandemic period samples were positive at this cutoff, we believe it to be valid.^7^ Furthermore, although the magnitude of the prevalence noted at each time period would change if a higher cutoff point were used, the noted trends and general conclusions from our study would still be the same (data not shown). Our study was focused specifically on pH1N1 in swine and humans. Our diagnostic approach was therefore focused on this virus and would not have detected evidence of other influenza virus infection in swine. Virus culture was performed only in eggs, limiting our ability to isolate viruses that grow better in cell culture. We did not attempt direct detection by PCR before virus isolation. This diagnostic approach and the lack of tissue samples in the 2nd post‐pandemic period may explain the low number of viruses isolated. We were able to perform whole‐genome sequencing on only two of the viruses from swine and two from humans collected in our study, and there were limited pH1N1 sequence data available from swine in Latin America in GenBank. Furthermore, given the low genetic diversity of the newly emerged pH1N1 virus,^1^ phylogenies of individual segments had a low resolution and relatively few bootstrap values >70, limiting the inference of detailed transmission dynamics. Finally, this study consisted of four serial cross‐sectional samplings, and therefore, it provided prevalence per each period that may have differed if other periods have been evaluated.^41^

Backyard farms provide ample opportunity for contact between humans, swine, wild and domestic birds, and a range of other animals.^22^, ^23^ This setting would thus seem to be especially suitable for the circulation of diverse strains of human as well as zoonotic influenza viruses, with the potential for virus coinfection and production of reassortants. Indeed, numerous reassortants between pH1N1 and other influenza viruses have been reported.^10^, ^16^, ^17^, ^18^, ^19^, ^20^, ^42^, ^43^, ^44^ Although the small number of animals on backyard farms and limited transference of animals between farms may limit the potential for virus transmission between swine, continued intensive surveillance for influenza virus reassortants in backyard farms is warranted.

## Addendum

Marc‐Alain Widdowson and John Barnes, U.S. Centers for Disease Control and Prevention, Atlanta, GA, U.S.A., involved in interpretation of the data and revising the intellectual content. Hugo Razuri and Maria C. Guezala, U.S. Naval Medical Research Unit No. 6, Lima, Peru, involved in interpretation of the data and critical writing of the manuscript.

## Disclosures

The views expressed in this article are those of the author and do not necessarily reflect the official policy or position of the Department of the Navy, Department of the Army, Department of Defense, Centers for Disease Control and Prevention, nor the U.S. Government. Several authors of this manuscript are military service members or employees of the U.S. government. This work was prepared as part of their official duties. Title 17 U.S.C. §105 provides that ‘Copyright protection under this title is not available for any work of the United States Government.' Title 17 U.S.C. §101 defines a U.S. government work as a work prepared by a military service member or employee of the U.S. government as part of that person's official duties. The authors declare they have no competing interests.

## Supporting information


**Figure S1.** Maximum‐likelihood phylogeny of PB2 gene sequence from A(H1N1)pdm09 viruses from Western Hemisphere, 2009–2011.

**Figure S2.** Maximum‐likelihood phylogeny of PB1 gene sequence from A(H1N1)pdm09 viruses from Western Hemisphere, 2009–2011.

**Figure S3.** Maximum‐likelihood phylogeny of PA gene sequence from A(H1N1)pdm09 viruses from Western Hemisphere, 2009–2011.

**Figure S4.** Maximum‐likelihood phylogeny of HA gene sequence from A(H1N1)pdm09 viruses from Western Hemisphere, 2009–2011.

**Figure S5.** Maximum‐likelihood phylogeny of NP gene sequence from A(H1N1)pdm09 viruses from Western Hemisphere, 2009–2011.

**Figure S6.** Maximum‐likelihood phylogeny of NA gene sequence from A(H1N1)pdm09 viruses from Western Hemisphere, 2009–2011.

**Figure S7.** Maximum‐likelihood phylogeny of MP gene sequence from A(H1N1)pdm09 viruses from Western Hemisphere, 2009–2011.

**Figure S8.** Maximum‐likelihood phylogeny of NS gene sequence from A(H1N1)pdm09 viruses from Western Hemisphere, 2009–2011. Virus sequences shown were collected from swine (blue with asterisk) and humans in Tumbes (black with asterisk), swine strains from the Western Hemisphere (red), and human strains (black) (see explanation in text). Bootstrap values >70 are included for key nodes, and tree is midpoint rooted for clarity only. GenBank accession numbers of the sequences used in this analysis can be found at http://www.ncbi.nlm.nih.gov/genomes/FLU/Database/nph-select.cgi.

## Appendix 1 Genetic relationship (based on percent nucleotide homogeneity of hemagglutinin and neuraminidase genes) between influenza A(H1N1)pdm09 viruses isolated from swine and humans in Tumbes, Peru, 2009–2011

**Table:**

Strain	A/sw/Peru/2010729235/2009 (swine)	A/sw/Peru/2010731246/2009 (swine)	A/sw/Peru/2010729232/2009 (swine)	A/Peru/2010731296/2009 (human)	A/Peru/2010731297/2009 (human)
Hemagglutinin					
A/sw/Peru/2010729235/2009 (swine)	100·0	99·9	99·8	99·8	99·6
A/sw/Peru/2010731246/2009 (swine)	99·9	100·0	99·7	99·7	99·5
A/sw/Peru/2010729232/2009 (swine)	99·8	99·7	100·0	99·9	99·7
A/Peru/2010731296/2009 (human)	99·8	99·7	99·9	100·0	99·7
A/Peru/2010731297/2009 (human)	99·6	99·5	99·7	99·7	100·0
Neuraminidase					
A/sw/Peru/2010729235/2009 (swine)	100·0	100·0	99·8	99·7	99·7
A/sw/Peru/2010731246/2009(swine)	100·0	100·0	99·8	99·7	99·7
A/sw/Peru/2010729232/2009 (swine)	99·8	99·8	100·0	99·9	99·9
A/Peru/2010731296/2009 (human)	99·7	99·7	99·9	100·0	99·9
A/Peru/2010731297/2009 (human)	99·7	99·7	99·9	99·9	100·0

## Appendix 2 Influenza virus strains and GenBank accession numbers used to create the phylogenetic tree in Figure 3

**Table:**

*N*	Accession	Strain	Host	Country
1	KM289084	A/swine/Peru/2010729235/2009 (H1N1)	Swine	Peru
2	KM289087	A/swine/Peru/2010729235/2009 (H1N1)	Swine	Peru
3	KM289085	A/swine/Peru/2010731246/2009 (H1N1)	Swine	Peru
4	KM289088	A/swine/Peru/2010731246/2009 (H1N1)	Swine	Peru
5	KM289086	A/swine/Peru/2010729232/2009 (H1N1)	Swine	Peru
6	KM289089	A/swine/Peru/2010729232/2009 (H1N1)	Swine	Peru
7	KM289090	A/Peru/2010731296/2009(H1N1)	Human	Peru
8	KM289092	A/Peru/2010731296/2009(H1N1)	Human	Peru
9	KM289091	A/Peru/2010731297/2009(H1N1)	Human	Peru
10	KM289093	A/Peru/2010731297/2009(H1N1)	Human	Peru
11	CY044256	A/swine/Argentina/SAGiles‐31215/2009(H1N1)	Swine	Argentina
12	JQ666845	A/swine/Brazil/1/2009(H1N1)	Swine	Brazil
13	JQ666855	A/swine/Brazil/11/2009(H1N1)	Swine	Brazil
14	JQ666856	A/swine/Brazil/12/2009(H1N1)	Swine	Brazil
15	JQ666857	A/swine/Brazil/13/2009(H1N1)	Swine	Brazil
16	JQ666860	A/swine/Brazil/16/2009(H1N1)	Swine	Brazil
17	JQ666861	A/swine/Brazil/17/2009(H1N1)	Swine	Brazil
18	JQ666846	A/swine/Brazil/2/2009(H1N1)	Swine	Brazil
19	JQ666847	A/swine/Brazil/3/2009(H1N1)	Swine	Brazil
20	JQ666848	A/swine/Brazil/4/2009(H1N1)	Swine	Brazil
21	JQ666849	A/swine/Brazil/5/2009(H1N1)	Swine	Brazil
22	JQ666850	A/swine/Brazil/6/2009(H1N1)	Swine	Brazil
23	JQ666851	A/swine/Brazil/7/2009(H1N1)	Swine	Brazil
24	HM569666	A/Argentina/07‐09GP/2009(H1N1)	Human	Argentina
25	HM569674	A/Argentina/08AR/2009(H1N1)	Human	Argentina
26	HM569682	A/Argentina/19527/2009(H1N1)	Human	Argentina
27	HM569690	A/Argentina/19618/2009(H1N1)	Human	Argentina
28	HM569698	A/Argentina/19656/2009(H1N1)	Human	Argentina
29	CY047776	A/Argentina/7649/2009(H1N1)	Human	Argentina
30	CY047784	A/Argentina/7785/2009(H1N1)/	Human	Argentina
31	CY047808	A/Argentina/7967/2009(H1N1)	Human	Argentina
32	CY047800	A/Argentina/7953/2009(H1N1)	Human	Argentina
33	CY047816	A/Argentina/7980/2009(H1N1)	Human	Argentina
34	CY047824	A/Argentina/8019/2009(H1N1)	Human	Argentina
35	CY047840	A/Argentina/8574/2009(H1N1)	Human	Argentina
36	CY047848	A/Argentina/8673/2009(H1N1)	Human	Argentina
37	CY047856	A/Argentina/8989/2009(H1N1)	Human	Argentina
38	CY047864	A/Argentina/8994/2009(H1N1)	Human	Argentina
39	CY047872	A/Argentina/9004/2009(H1N1)	Human	Argentina
40	CY047880	A/Argentina/9180/2009(H1N1)	Human	Argentina
41	CY047888	A/Argentina/9333/2009(H1N1)	Human	Argentina
42	CY047896	A/Argentina/9384/2009(H1N1)	Human	Argentina
43	CY047920	A/Argentina/9579/2009(H1N1)	Human	Argentina
44	CY047952	A/Argentina/9705/2009(H1N1)	Human	Argentina
45	CY047968	A/Argentina/9721/2009(H1N1)	Human	Argentina
46	CY083085	A/Bogota/WRAIR0442T/2009(H1N1)	Human	Colombia
47	CY120722	A/Brazil/AVS03/2009(H1N1)	Human	Brazil
48	CY120730	A/Brazil/AVS04/2009(H1N1)	Human	Brazil
49	CY120770	A/Brazil/AVS05/2009(H1N1)	Human	Brazil
50	CY120738	A/Brazil/AVS06/2009(H1N1)	Human	Brazil
51	CY120746	A/Brazil/AVS07/2009(H1N1)	Human	Brazil
52	CY075162	A/Chile/158/2009(H1N1)	Human	Chile
53	CY075178	A/Chile/1586/2009(H1N1)	Human	Chile
54	CY075186	A/Chile/1598/2009(H1N1)	Human	Chile
55	CY075194	A/Chile/1599/2009(H1N1)	Human	Chile
