# Supplementary figures and images for: Transmission dynamics of pandemic influenza A(H1N1)pdm09 virus in humans and swine in backyard farms in Tumbes, Peru
Source: Influenza Other Respir Viruses. 2015 Dec 11;10(1):47–56. doi: 10.1111/irv.12329 (PMC4687498; doi:10.1111/irv.12329)

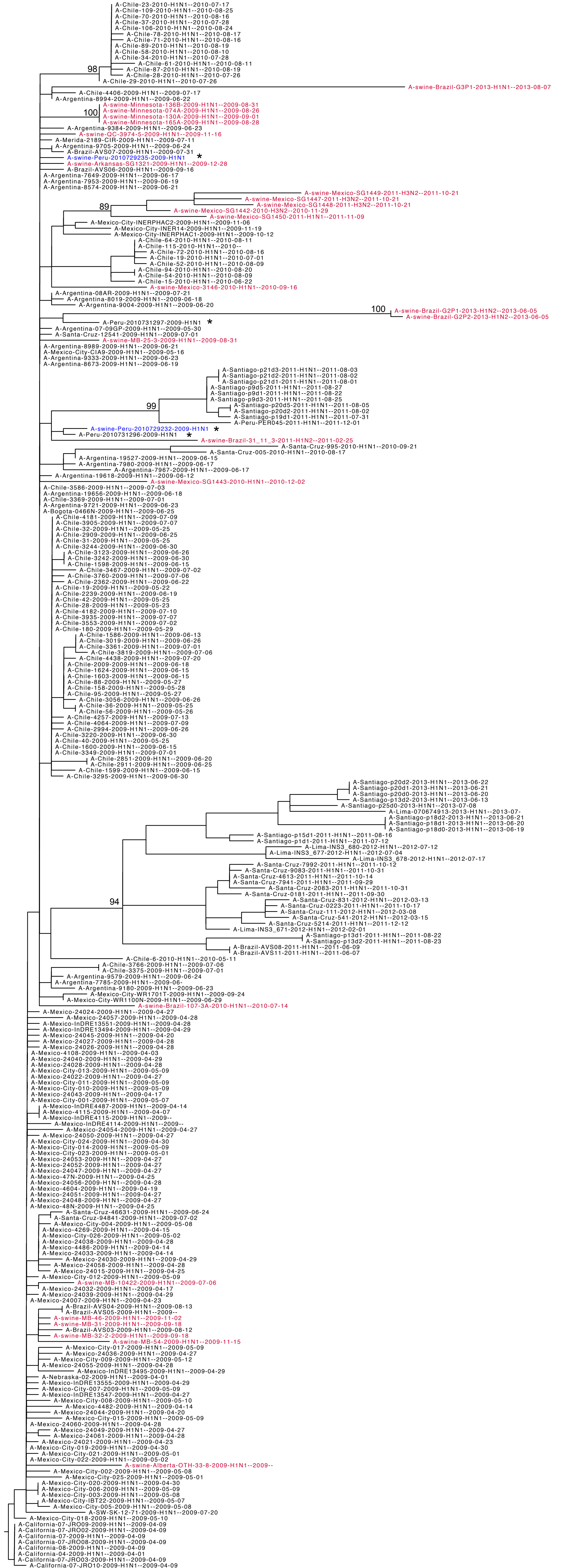

Supplement: Supplementary file 1 — Figure S1. Maximum‐likelihood phylogeny of PB2 gene sequence from A(H1N1)pdm09 viruses from Western Hemisphere, 2009–2011. [file IRV-10-47-s001.pdf]

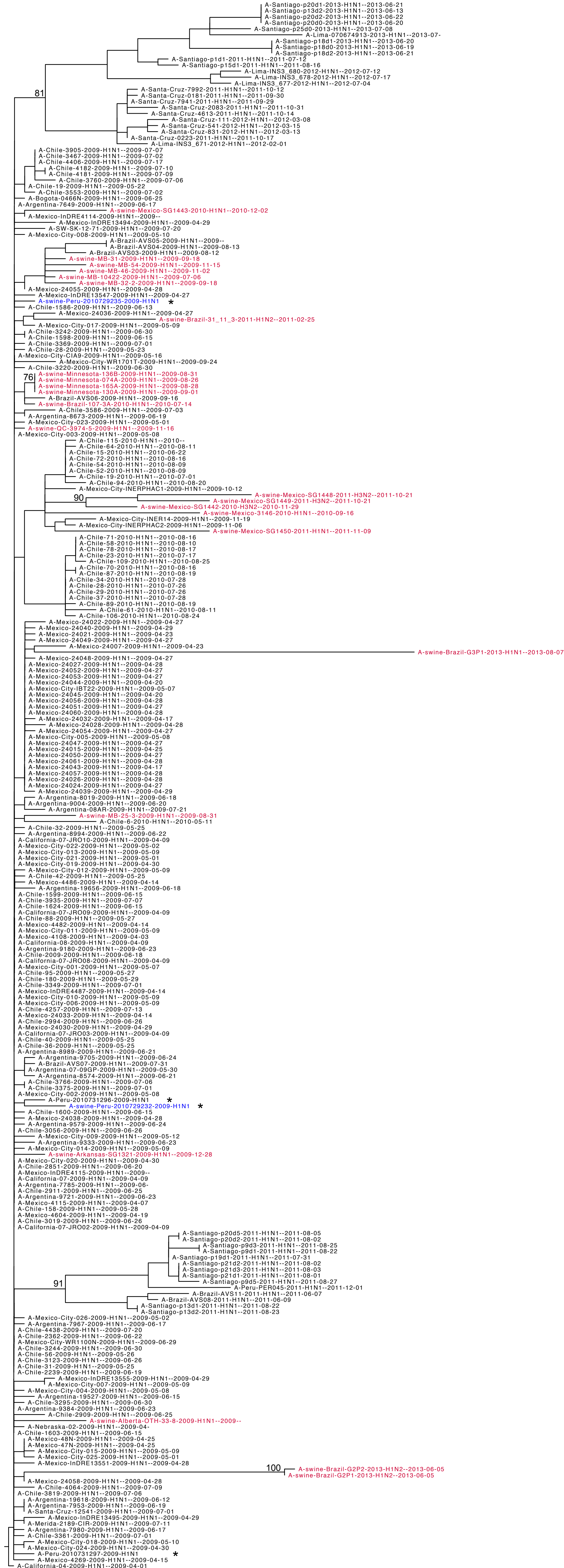

0 0020

Supplement: Supplementary file 2 — Figure S2. Maximum‐likelihood phylogeny of PB1 gene sequence from A(H1N1)pdm09 viruses from Western Hemisphere, 2009–2011. [file IRV-10-47-s002.pdf]

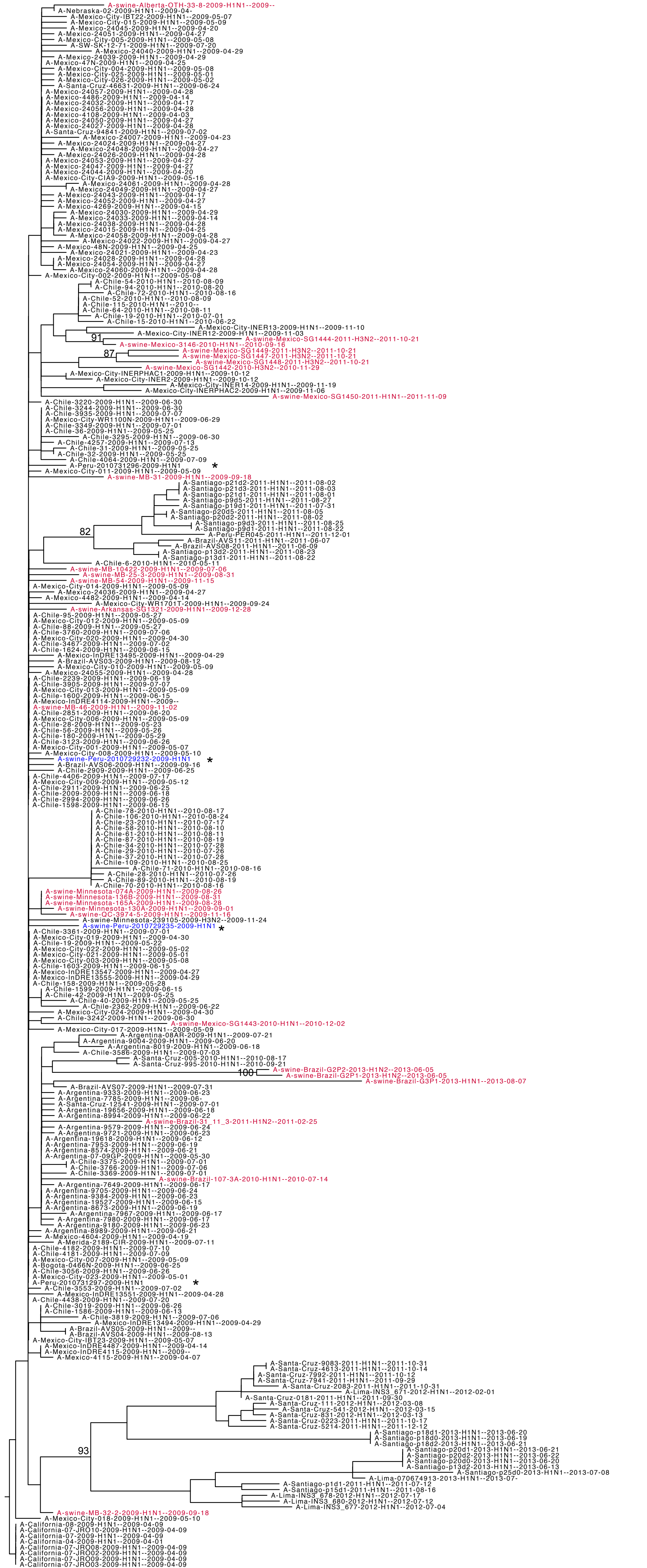

Supplement: Supplementary file 3 — Figure S3. Maximum‐likelihood phylogeny of PA gene sequence from A(H1N1)pdm09 viruses from Western Hemisphere, 2009–2011. [file IRV-10-47-s003.pdf]

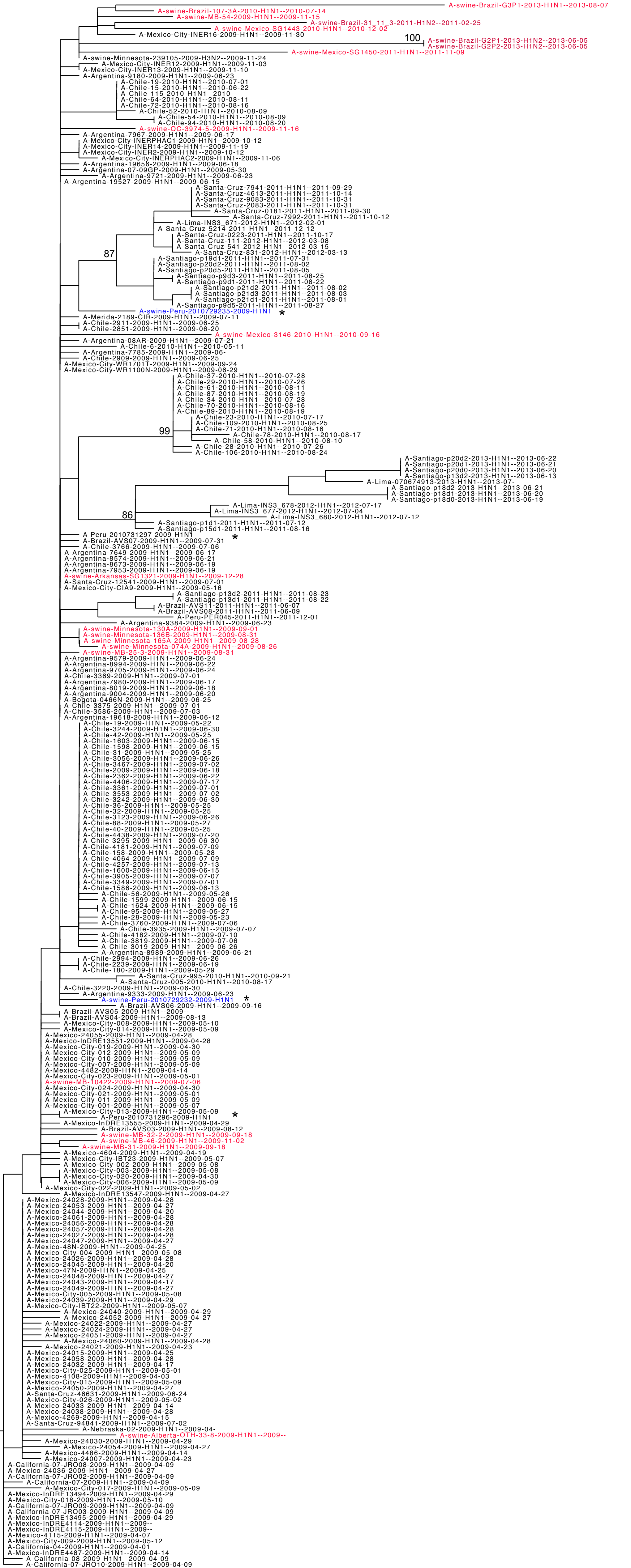

Supplement: Supplementary file 5 — Figure S5. Maximum‐likelihood phylogeny of NP gene sequence from A(H1N1)pdm09 viruses from Western Hemisphere, 2009–2011. [file IRV-10-47-s005.pdf]

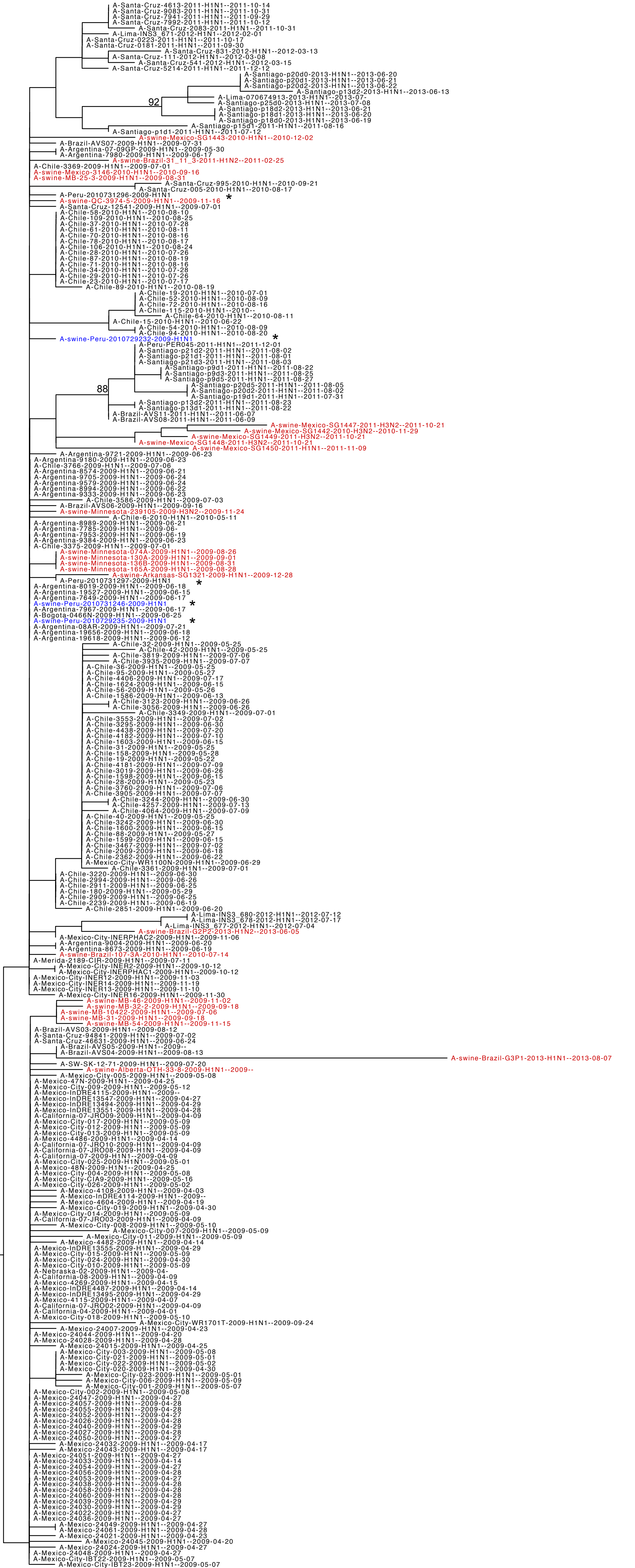

Supplement: Supplementary file 7 — Figure S7. Maximum‐likelihood phylogeny of MP gene sequence from A(H1N1)pdm09 viruses from Western Hemisphere, 2009–2011. [file IRV-10-47-s007.pdf]

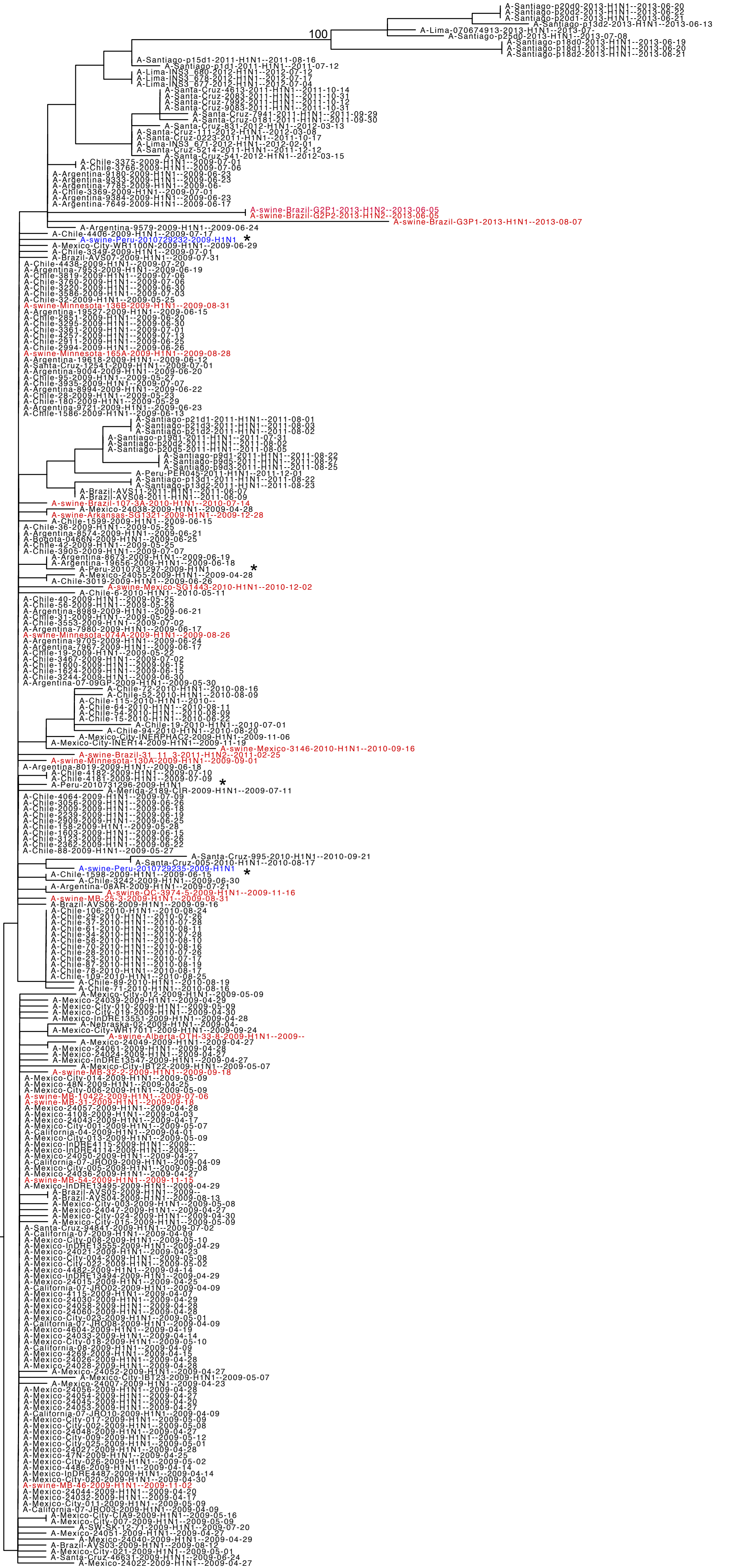

Supplement: Supplementary file 8 — Figure S8. Maximum‐likelihood phylogeny of NS gene sequence from A(H1N1)pdm09 viruses from Western Hemisphere, 2009–2011. Virus sequences shown were collected from swine (blue with asterisk) and humans in Tumbes (black with asterisk), swine strains from the Western Hemisphere (red), and human strains (black) (see explanation in text). Bootstrap values >70 are included for key nodes, and tree is midpoint rooted for clarity only. GenBank accession numbers of the sequences used in this analysis can be found at http://www.ncbi.nlm.nih.gov/genomes/FLU/Database/nph-select.cgi. [file IRV-10-47-s008.pdf]
