# Supplementary material for: Transmission dynamics of pandemic influenza A(H1N1)pdm09 virus in humans and swine in backyard farms in Tumbes, Peru
Source: Influenza Other Respir Viruses. 2015 Dec 11;10(1):47–56. doi: 10.1111/irv.12329 (PMC4687498; doi:10.1111/irv.12329)
Supplement: Supplementary file 4 — Figure S4. Maximum‐likelihood phylogeny of HA gene sequence from A(H1N1)pdm09 viruses from Western Hemisphere, 2009–2011. [file IRV-10-47-s004.pdf]

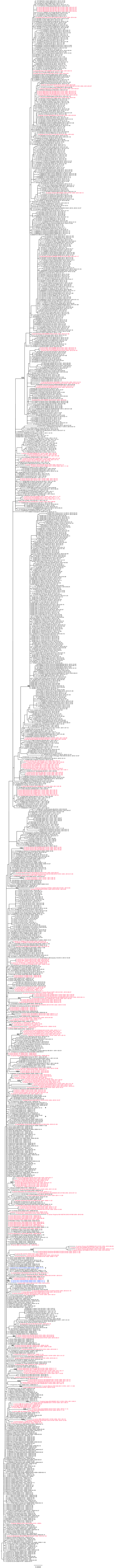
0.0040
